# Isolation and purification of beta-lactoglobulin from cow milk

**DOI:** 10.14202/vetworld.2015.621-624

**Published:** 2015-05-15

**Authors:** Ranjit Aich, Subhasis Batabyal, Siddhartha Narayan Joardar

**Affiliations:** 1 Department of Veterinary Biochemistry, Faculty of Veterinary and Animal Sciences, West Bengal University of Animal and Fishery Sciences, Belgachia - 700 037, Kolkata, India; 2 Department of Veterinary Biochemistry, College of Veterinary Science and Animal Husbandry, Mhow - 453 446, Madhya Pradesh, India; 3 Department of Veterinary Microbiology, Faculty of Veterinary and Animal Sciences, West Bengal University of Animal and Fishery Sciences, Belgachia - 700 037, Kolkata, India

**Keywords:** beta-lactoglobulin, ion-exchange chromatography, milk protein intolerance, sodium dodecyl sulfate polyacrylamide gel electrophoresis, whey protein

## Abstract

**Aim:**

The present study was undertaken to standardize a convenient method for isolation and purification of β-lactoglobulin (β-lg) from cow milk keeping its antigenicity intact, so that the purified β-lg can be used for detection of cow milk protein intolerance (CMPI).

**Materials and Methods:**

Raw milk was collected from Gir breed of cattle reared in Haringhata Farm, West Bengal. Milk was then converted to skimmed milk by removing fat globules and casein protein was removed by acidification to pH 4.6 by adding 3 M HCl. β-lg was isolated by gel filtration chromatography using Sephacryl S-200 from the supernatant whey protein fraction. Further, β-lg was purified by anion-exchange chromatography in diethylaminoethyl-sepharose. Molecular weight of the purified cattle β-lg was determined by 15 percent one-dimensional sodium dodecyl sulfate-polyacrylamide gel electrophoresis and was analyzed by gel documentation system using standard molecular weight marker.

**Results:**

The molecular weight of the purified cattle β-lg was detected as 17.44 kDa. The isolated β-lg was almost in pure form as the molecular weight of purified β-lg monomer is 18kDa.

**Conclusion:**

The study revealed a simple and suitable method for isolation of β-lg from whey protein in pure form which may be used for detection of CMPI.

## Introduction

Milk whey contains a heterogeneous group of proteins that can be derived from blood or synthesized in the mammary gland. Whey protein is widely used as a food ingredient and as an additive to improve the texture and quality of food and by providing amino acids required by the young animal. Beta-lactoglobulin (β-lg), a major whey protein found in the milk of cows and other ruminants is considered as a milk allergen [1-4]. However, it is not an endogenous substance in human, rodents, and lagomorph’s milk. β-lg, a major protein that accounts for approximately 10-15% of total milk proteins and 58% of whey protein, exists at the normal pH of bovine milk as a dimer with a molecular weight of 36 kDa [5]. It is a single chain polypeptide of 18 kDa comprising of 162 amino acid residues. The complete amino acid sequence of β-lg has been reported and genetic variation in amino acid sequence has been identified [6]. It could have a role in metabolism of phosphate in the mammary gland [7] and the transport of retinol and fatty acids in the gut [8], or in the transfer of passive immunity to the newborn [9]. β-lg, the principal whey protein, possesses multiple sites for binding ligands [10-13]. Ligand-binding properties of β-lg are well documented, but the subsequent biological functions are still unclear [14], although it has been widely studied for its functional properties [15].

β-lg is a mild antioxidant whose potency is less than that of vitamin E [16]. The conversion of the β-lg monomer to dimer was responsible, in part, for the mode of action in protecting low-density lipoproteins against copper-induced oxidation. Cross-linking the free thiol groups of β-lg by heating (100°C for 2 min), or chemically modifying the β-lg by carboxymethylation to block the thiol groups resulted in a substantial loss of antioxidant activity [16]. Proteolytic digestion of bovine β-lg by trypsin yielded four peptide fragments that have bactericidal effects against the Gram-positive bacteria only [17].

β-lg seems to be quite resistant to gastric digestion *in-vivo* and apparently remains mostly intact after it passes through the stomach [18]. Better understanding of the underlying mechanisms of food hypersensitivity reactions has always necessitated studying antigenic and molecular characteristics of food antigens. To do this, it is important to have the antigens in the pure form. Having these antigens, on the other hand, will allow us to determine specific antibodies. The present study was undertaken with the objective to standardize a suitable and simple procedure of purification of β-lg from cow milk.

## Materials and Methods

### Ethical approval

As per CPCSEA guidelines, study involving clinical samples does not require approval of Institute Animal Ethics Committee.

### Preparation of whey

Raw milk was collected from Gir breed of cattle of Haringhata Farm, West Bengal. Fresh raw milk after collection was filtrated with quadrilayer gauze in order to remove impurities. Then the milk was centrifuged at 3000 rpm for 30 min at 4°C and the top fat layer was removed by a spatula. The skimmed milk was used for the preparation of whey protein as per Caessens et al. [19]. Briefly, the skimmed milk was acidified to pH 4.6 by adding 3 M HCl slowly. Then the solution was incubated for 30 min at 40°C and caseins were removed by centrifugation at 8000 rpm for 15 min at 4°C and the supernatant was poured over glass wool. The pH of the acidic whey fraction was raised to pH 7.2 with 1N NaOH, and the material was subsequently centrifuged for 15 min at 10°C at 8000 rpm. The supernatant was filtered and this material was referred as a whey protein fraction (WPF). The concentration of cow whey proteins was determined by Lowry’s method [20] and aliquots were stored at −20°C until used.

### Isolation of β-lg

To isolate β-lg from cow WPF, Sephacryl S-200 (Sigma-Aldrich Co., USA.) was degassed and packed into the column (43.0 cm × 2.0 cm) [21]. The matrix was equilibrated with 0.02 M phosphate buffer, pH 6.8 and the sample containing 15.0 mg of protein was loaded. The elution was carried out with equilibrating buffer by using 3 ml of the fraction at a flow rate of 0.20 ml/min with 0.02 M phosphate buffer, pH 6.8. The absorbance of the fractions was monitored by taking the absorbance at 280 nm in a UV/VIS spectrophotometer 119 (Systronics). The absorbance values were plotted against fraction number and obtained one major Peak A (test tube no. 30, 31, 32, 33, 34, 35) and two minor peaks i.e., Peak B (test tube no. 39) and Peak C (test tube no. 42, 43, 44). Protein fractions of Peak A, Peak B, and Peak C were pooled, concentrated by sucrose and named S-I, S-II, and S-III, respectively. The protein concentration of S-I, S-II, and S-III was determined [20] and were preserved at 20°C in aliquots for further use.

### Purification of β-lg

Anion-exchange chromatography was performed on diethylaminoethyl (DEAE) - Sepharose gel column (13 cm × 1.5 cm). The loading buffer, equilibrating buffer, and elution buffer were as follows: 0.02 M phosphate buffer, pH 6.8; 0.02 M phosphate buffer, pH 6.8; 0.02 M phosphate buffer containing 0-0.5 M NaCl, pH 6.8, respectively. The matrix was equilibrated with equilibrating buffer, and the sample (S-I) collected after gel filtration of WPF containing highest protein was loaded. Total 7.40 mg of protein of S-I was loaded. The bound proteins were eluted at a linear gradient by using elution buffer with flow-rate and fraction volume being 0.30 ml/min and 3 ml, respectively. Protein fractions of 1^st^ peak (named D) at 0.2 M NaCl (test tube no. 9, 10, 11) and that of 2^nd^ peak (named E) at 0.3 M NaCl (test tube no. 20, 21) were pooled and concentrated by sucrose. Finally, the concentration of the pooled proteins (named S-IV and S-V, respectively) was determined [20] and preserved at –20°C in aliquots for further use. Purity of S-IV and S-V was checked by one-dimensional 15% sodium dodecyl sulfate polyacrylamide gel electrophoresis (SDS-PAGE) in vertical slab gel electrophoresis chamber (AE-6200) with power supply (ATTO Corporation, Japan) as per Laemmli [22], with some modification under denaturing and reducing condition. Molecular weights of purified samples were analyzed by gel documentation system (Bio-Rad) using medium range protein markers (PMW-M, GENEI) after staining with Coomassie brilliant blue R250.

## Results and Discussion

**Figure-1:**
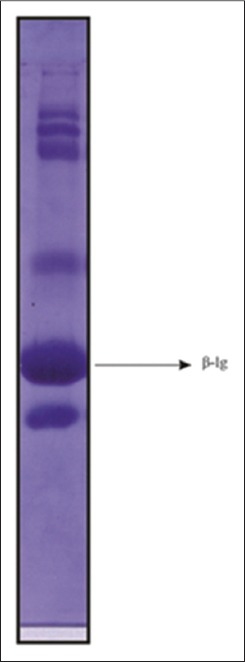
Sodium dodecyl sulfate polyacrylamide gel electrophoresis (15% gel) of crude whey protein from cattle milk.

**Figure-2:**
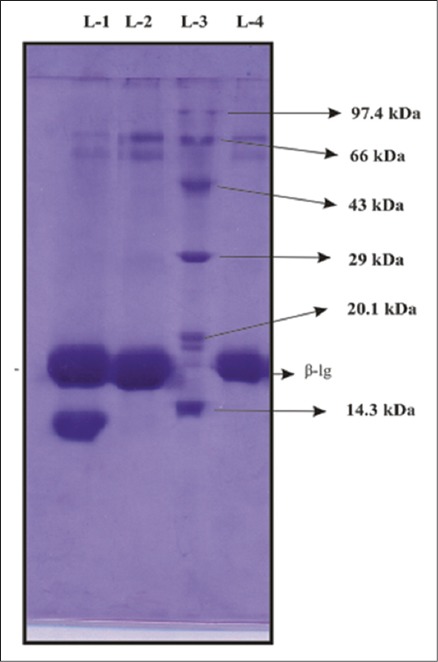
Sodium dodecyl sulfate-polyacrylamide gel electrophoresis analysis of different fractions of cattle whey protein obtained after gel filtration chromatography using Sephacryl S-200, L-1: S-III, L-2: SII, L-3: M-Standard protein molecular weight marker (medium range), L-4: S-I.

The total concentration of cattle whey proteins was about 7.50 mg/ml. SDS-PAGE of WPF (Figure-1) isolated from cow milk showed a similar pattern of migration with 6 major bands after staining with Coomassie brilliant blue R250. β-lg from cattle WPF was isolated by gel filtration chromatography using Sephacryl S-200 (Figure-2) and the protein concentration of S-I sample was highest, i.e., 3.70 mg/ml. Several methods have been reported for isolation of β-lg from whey, but most of them are expensive and do not give high yields. Among the methods used to separate it from whey are precipitation at low pH and peptic hydrolysis followed by selective membrane filtration [23], Bio-Gel P10 column at pH 3.0 [24], cat-ion exchange selective absorption process [25], the monolithic DEAE convective interaction media analytical column (CIMac DEAE) [26].

**Figure-3:**
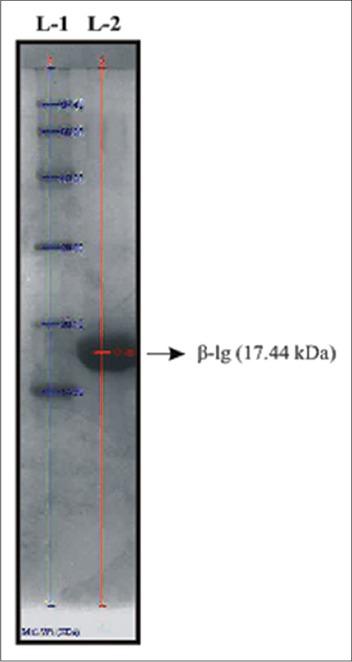
Sodium dodecyl sulfate-polyacrylamide gel electrophoresis analysis of purified cattle β-lactoglobulin by gel documentation system obtained after anion-exchange chromatography of gel chromatographed fraction (S-I) using DEAE-Sepharose, L-1: Molecular weight marker ranges 14.3-97.4 kDa, L-2: S-IV (Purified cattle β-lactoglobulin).

Purification of β-lg from gel filtration samples (S-I) of cow whey protein was done by anion-exchange chromatography on DEAE-Sepharose. The DEAE-Sepharose chromatography results in the fractions S-IV and S-V. The sample S-IV showed single band of purified protein of 17.44 kDa (Figure-3) as analyzed by gel documentation system (Bio-Rad) using standard molecular weight marker (range 14.3-97.4 kDa) in 15% one-dimensional SDS-PAGE. The present finding correlates the earlier observations where MALDI-TOF spectrum of the purified bovine β-lg clearly identified A and B types with molecular masses of 18.371 kDa and 18.284 kDa, respectively [27]. Molecular weight of the purified buffalo β-lg was 18.05kDa as assessed by the gel documentation system in 15 percent one-dimensional SDS-PAGE [28] previously, electrophoretic analysis of purified cattle β-lg was performed in SDS-PAGE and the molecular weights were estimated at 34,000 Da for β-lg dimmer and 16,000 Da for β-lg monomer [21]. In contrary, the molecular weight of β-lg isolated by ammonium sulfate precipitation and purified by preparative scale gel filtration was found to be 18,400 by SDS-PAGE analysis and 36,800 by fplc-GF analysis [23].

Taken together, β-lg could be isolated in almost pure form (seen as a single band in gel). Any basic or applied study pertaining to bio-molecule requires purity of the starting material. As such these simple steps encompassing chromatographic techniques might be a useful means in getting β-lg molecule in pure form that might be useful in the next step to mitigate cow milk protein intolerance (CMPI).

## Conclusion

The study revealed a simple and suitable method for isolation of β-lg from whey protein in almost pure form that might be exploited for any basic and applied study related to CMPI and this method could possibly be adapted for other proteins as well.

## Authors’ Contributions

RA and SNJ implemented the study design and carried out the experiment. SB and SNJ analyzed the data. RA and SB drafted and revised the manuscript. All authors read and approved the manuscript.
